# Evaluating the Efficacy of Resveratrol-Containing Mouthwash as an Adjunct Treatment for Periodontitis: A Randomized Clinical Trial

**DOI:** 10.1055/s-0044-1788686

**Published:** 2024-09-09

**Authors:** Sura A. Mohammed, Hadeel Mazin Akram

**Affiliations:** 1 Department of Periodontology, College of Dentistry, University of Baghdad, Baghdad, Iraq

**Keywords:** resveratrol, periodontitis, mouthwash

## Abstract

**Objectives** To evaluate the effectiveness of resveratrol mouthwash as an adjunct to nonsurgical periodontal treatment of periodontitis.

**Materials and Methods** This study was a randomized, double-blind clinical trial study. The study included 57 participants with periodontitis. Clinical parameters (plaque index [PI], bleeding on probing [BOP], probing pocket depth [PPD], and clinical attachment loss [CAL]) were examined at the baseline visit, after 7 days, and after 30 days of using resveratrol mouthwash as an adjunct to nonsurgical periodontal treatment. The salivary levels of (interleukin [IL]-6) and RANKL (receptor activator of nuclear factor-kappa B ligand) were measured and compared before and after treatment. The participants answered the visual analog scale-based assessment questionnaire at the last visit.

**Statistical Analysis** A one-way ANOVA (analysis of variance) test was used to compare the means of multiple groups (test, positive control, negative control) at baseline and after treatment. A paired *t* -test was also used to compare the means of a single group before and after treatment. In addition, Tukey's multiple comparisons test was used to identify specific pairwise differences between the three groups after finding significant differences with ANOVA. The Chi-square test was also used to compare the distribution of categorical variables like sex between the groups.

**Results** All interventions significantly reduced PI, BOP, PPD, and CAL, but resveratrol and chlorhexidine had a higher significant effect than placebo except for CAL without a significant difference between them. All mouthwashes significantly reduced the salivary concentration of IL-6. However, resveratrol and chlorhexidine had a significantly higher effect than placebo, while the concentration of RANKL was decreased in all groups without a significant difference between them. The participants' responses to the mouthwash questionnaire showed that resveratrol and chlorhexidine had the same feedback without significant differences.

**Conclusion** Resveratrol-containing mouthwash could be used as an alternative to chlorhexidine as an adjunct to nonsurgical periodontal treatment of periodontitis.

## Introduction

Periodontitis is a multifactorial progressive disease characterized by the progressive destruction of periodontal tissue due to dysbiotic bacteria, host immunological response, environmental variables, and subject genetic susceptibility. 1 The penetration of bacteria into the gingiva triggers an immunological response from the host and initiates inflammation of the gingiva. 2 3 This inflammation then leads to the destruction of the underlying periodontal tissues and the loss of alveolar bone. 4 The primary objectives of periodontal treatment are to reduce inflammation, prevent deep tissue invasion, and establish an environment suitable for periodontal tissue healing and regeneration. 5

Standard periodontal therapy techniques include nonsurgical periodontal (NSP) treatment. This approach typically results in improvements in clinical periodontal parameters, a considerable reduction in probing pocket depth (PPD), and an increase in clinical attachment level, especially in deeper areas. 6 7

Antibiotics and nonsteroidal anti-inflammatory drugs have been considered adjunct therapies for periodontal treatment. Nevertheless, these medications are associated with antibiotic resistance, gastric intolerance, and systemic problems. 8 Therefore, phytochemicals can be a safer and more effective pharmaceutical alternative. 9 Researchers have found that phytotherapeutic agents can suppress bacteria that lead to periodontal diseases; numerous studies have concentrated on the antibacterial influences of natural herbs against *Porphyromonas gingivalis* and attempted to use alternatives to antibiotics and antiseptics for periodontal therapy. 10

Resveratrol, also known as RSV or 3,5,4′-trihydroxy- *trans* -stilbene, is a phytoalexin synthesized by different plants and is highly present in the skin of red grapes and peanuts. It is frequently used as a nutritional supplement to enhance metabolic problems. 11 Resveratrol (RSV) has beneficial properties such as antioxidant activity, promoting the endothelial generation of nitric oxide, reducing lipid levels, inhibiting platelet aggregation, and suppressing vascular inflammation. 12 13 The beneficial properties of RSV have been attributed to its possible application in treating hypertension, 14 type 2 diabetes, 15 cardiovascular diseases, 16 ischemic stroke, 17 atrial fibrillation and heart failure, 18 hepatic steatosis, 19 cancer, 20 and metabolic syndrome. 21

RSV is considered a potential medical agent for preventing and treating inflammatory disorders. 22 By effectively managing inflammation, RSV can prevent the progress of periodontal disease. 23 RSV has anti-biofilm and antibacterial properties, specifically targeting inflammatory and adhesive markers. 24 It seems that RSV could be beneficial as a supplementary treatment for those with periodontitis. 25

Furthermore, to enhance the assessment of the immunomodulatory properties of RSV, they analyzed the cytokine profile, specifically focusing on the release of interleukin (IL)-6, which is known for its pro-inflammatory actions 26 27 and was increased in patients with periodontitis. 28 29 30 IL-6, at high concentrations, mainly activates mature osteoclasts. Additionally, research has shown that IL-6 is linked to the release and activation of Matrix metalloproteinases (MMPs), which can lead to pathological extracellular matrix degradation in periodontitis patients. 31

In addition, studies have identified that osteoclastic bone damage during periodontitis depends on the RANKL (receptor activator of nuclear factor-kappa B ligand) of osteoblastic cells and periodontal ligament cells. During periodontal inflammation, immune cells send essential signals that cause RANKL to be produced. 32 RANKL governs the proliferation, activation, and differentiation of osteoclasts, which leads to the loss of alveolar bone. 33 Previous studies revealed that the therapy with RSV reduced alveolar bone loss and suggested that the presence of RSV may suppress the production of RANKL and inhibit bone collapse. 34

To the best of our knowledge, no previous studies have been conducted on treating periodontitis with RSV mouthwash as an adjunct to NSP treatment.

This study aimed to evaluate the effectiveness of RSV mouthwash as an adjunct to NSP treatment of periodontitis in comparison to a positive control (chlorhexidine [CHX]) and a negative control (placebo) and to evaluate the change in the salivary level of IL-6 and RANKL.

The study's null hypothesis is that RSV mouthwash is ineffective as an adjunct to NSP therapy for the treatment of periodontitis.

## Materials and Methods

### Study Design

This research was a double-blind, randomized clinical trial conducted at the Teaching Clinics Department of Periodontics, University of Baghdad, College of Dentistry, from January 2023 to August 2023. The study protocol obtained ethical approval (Ref. No.: 744, Date: 28 December 2022) from the Ethics Committee, College of Dentistry, University of Baghdad. The study protocol was registered in ClinicalTrials.gov (NCT05874882) ( Supplementary Material , available in the online version only).

All potential candidates were given informed consent forms to sign and were provided with a thorough description of the study's aims and objectives, and they were free to withdraw from the study at any time.

### Sample Size

The sample size was estimated using data obtained from previous research. 35 In successful active periodontal treatment using manual instrumentation, periodontal pockets were reduced from 7.64 ± 1.76 to 5.68 ± 2.3. Using G*Power software, a sample size of 17 was estimated to reject the study's null hypothesis at probability 0.05 and power of 0.80. To avoid a possible 20% dropout of patients during follow-up, 20 patients in each group were recruited in this study ( *n* = 20).

### The Inclusion Criteria

The inclusion criteria included: systemically healthy patient age >18 years old, with periodontitis (interdental clinical attachment loss [CAL] at ≥2 nonadjacent teeth, or buccal or oral CAL ≥3 mm with pocketing ≥4 mm is detectable at ≥2), which includes stage II (bone loss involving coronal 1/3 of the root) or stage III (bone loss involving the middle 1/3 of the root). The periodontitis cases should be unstable periodontitis (PPD ≥4 mm with BOP or PPD >5 mm with or without BOP), 36 the patient's body mass index falls within the normal healthy range (18.5–24.9), should have a minimum of 20 teeth, it is necessary to have not taken antibiotics and anti-inflammatory medications for the last 3 months, and the periodontal pocket depth should be 4 to 7 mm. The patient should have at least two pockets.

### The Exclusion Criteria

The exclusion criteria included patients wearing fixed prostheses, patients with overhang filling tooth or tooth anomalies, individuals with chronic disease, immunocompromised patients, pregnant and lactating women, those using contraceptives, those using mouthwash, those with a history of hypersensitivity to any product used in this study, smokers, or alcoholics.

### Randomization and Blinding

**Fig. 1:**
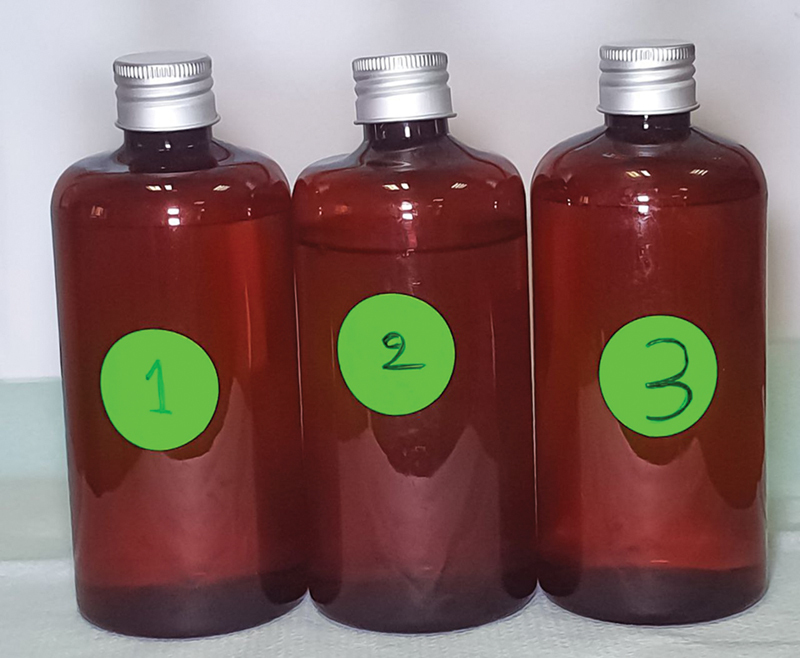
Blinding the mouthwash by number-coded.

Each participant had an equal chance of being assigned to the intervention sequence, which was determined randomly by block randomization. RSV (test), 0.12% CHX (positive control), and distilled water with flavors (negative control) were used in this study. The mouthwashes were stored in matching opaque bottles and assigned subsequent number codes (1, 2, and 3) by a dentist not associated with this study ( Fig. 1 ).

### Study Groups

*Test group:* they used Oroxil mouthwash. The essential ingredient is the nano-technology-boosted RSV with the essential oil of the wild Thyme herb (Logidex Srl, Italy).

*Positive control group:* they used CHX mouthwash containing 0.12% chlorhexidine digluconate (kin, Ireland).

*Negative (placebo) group:* the placebo mouth rinse was composed of distal water, food coloring, and flavor.

All mouthwash bottles were similar in appearance and used for 60 seconds, twice daily for a period of 4 weeks. 37 38

### Clinical Procedure

*First visit (baseline)* : full medical and dental history was taken from each participant, and samples of unstimulated saliva were acquired from the participants. Dental impressions were taken to fabricate the stent (which is used in recording clinical periodontal parameters to minimize error and over- and underestimation of measurements during the clinical trials). 39 Clinical periodontal parameters (full mouth plaque index [PI], bleeding on probing [BOP], PPD, and CAL) were measured by a single blinded examiner using a periodontal probe (UNC15). Supragingival professional mechanical plaque removal (PMPR) was performed by the same examiner for all patients using an ultrasonic device (WOODPECKER UDS-K, India). All of the patients received tooth-brushing instructions for the Modified Bass technique and brushed their teeth twice a day with a toothbrush (Oral B indicator, Ireland) and toothpaste (Oral B Pro-Expert, Germany) given for 4 weeks.

*Second visit (after 7 days from the first visit)* : measurement of clinical periodontal parameters (PI, BOP, PPD, and CAL) followed by full mouth root surface debridement (RSD). The participants were provided with coded bottles containing the interventions. They were instructed to rinse their mouths with 10 mL of undiluted mouthwash twice daily for 60 seconds, specifically 30 minutes after brushing their teeth. The individuals were instructed to avoid consuming food or drink for 30 minutes following rinsing and contacted by phone daily with participants during the intervention period to assess general health conditions, mouthwash tolerability, and potential adverse events.

*Third visit (after 4 weeks from root planning)* : collection of saliva samples followed by measurement of clinical periodontal parameters (PI, BOP, PPD, and CAL), and then assessed treatment adherence by requesting and weighting the bottle at the end of the trial and saw if there are remaining mouth wash in a bottle.

### Clinical Assessment

Clinical periodontal parameters, including PI, BOP, PPD, and CAL were assessed by a single examiner using University of North Carolina-15 (UNC-15) probes, which are color-coded at every millimeter demarcation.

The PI was measured by using the O'Leary PI. 40 A suitable disclosing solution (Bioclear matrix) was painted on all exposed tooth surfaces at the initial appointment. After the patient rinsed, the examiner used an explorer or probe tip to examine each stained surface for soft accumulations. After all teeth were examined and scored, the mean was calculated by dividing the number of plaque surfaces by the total number of available surfaces multiplied by 100%.

BOP was measured by gently inserting the periodontal probe at the six surfaces of all teeth to the depth of the gingival sulcus\periodontal pocket and then removed coronally and waiting for 30 seconds to observe the presence or absence of bleeding (0 = no bleeding, 1 = presence of bleeding). 41

PPD is defined as a millimeter distance between the gingival margin to the most apical penetration of the periodontal probe inserted into the periodontal pocket (probing force not exceeding 0.25 N). 41

**Fig. 2:**
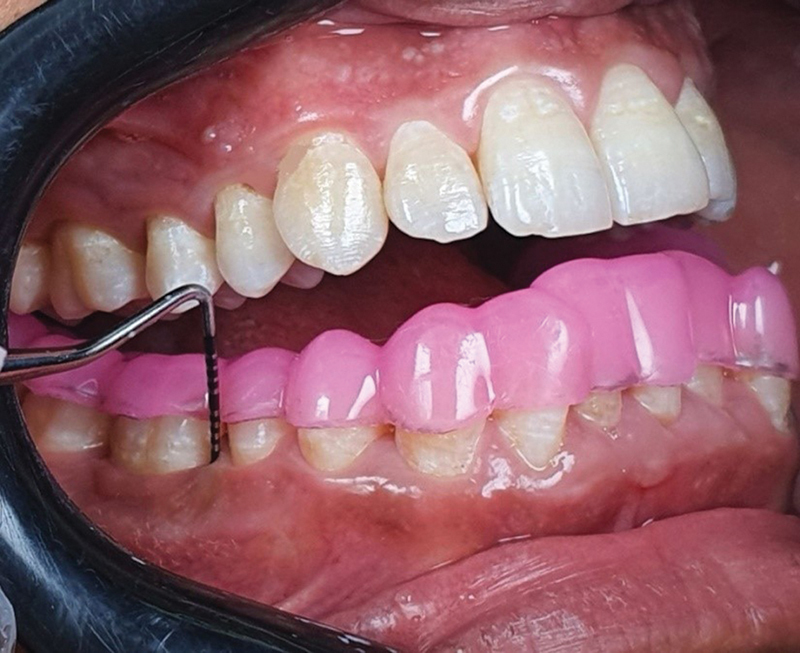
Acrylic stent was used to determine clinical attachment level.

Assessment of CAL was done by measuring six surfaces of each tooth except third molars by using a manual periodontal probe and custom-made acrylic stent with vertical groove. The periodontal probe was inserted gently parallel to the long axis of the tooth until resistance was noted, and the nearest millimeter was recorded from the lower border of the groove on the occlusal stent as a reference point to the base of the pocket ( Fig. 2 ).

### Calibration

The examiner's accuracy and consistency in measuring clinical periodontal parameters (PI, BOP, PPI, and CAL) were evaluated through inter- and intra-examiner calibration. Inter-examiner calibration scores were documented by the researcher with assistance from the supervisor. For intra-examiner calibration, the researcher measured the periodontal parameters of five subjects twice, with a 2-hour interval between measurements. This process was conducted to standardize and align the measurements, aiming to achieve an acceptable level of agreement, indicated by a kappa test score above 0.75 and an interclass coefficient above 90% for all clinical parameters.

### Salivary Sample Collection

Saliva samples were collected from patients at baseline visits before clinical examination and after 4 weeks of root planing. Subjects were asked to refrain from eating and drinking for 1 to 2 hours before the saliva collection. Unstimulated whole expectorated saliva (5 mL) was collected from each subject between 09:00 and 11:00 a.m. according to a modification in the method described. 42 Collected samples were placed immediately on ice and then frozen at −20°C until analysis by ELISA.

### Salivary Biomarker Analysis

All saliva samples were thawed to room temperature before the experimental procedures and centrifuged at 1,000 × g for 20 minutes to eliminate cellular debris. Commercially available ELISA kits (SHANGHAI YEHUA Biological Technology Co., Ltd.) were used to measure the concentration of salivary IL-6 and RANKL. The procedure was conducted following the manufacturer's instructions for each kit.

### Visual Analog Scale Questionnaire

Each participant answered a visual analog scale (VAS) questionnaire at the end of the study to evaluate the intervention. All subjects received a questionnaire using a VAS designed to assess their attitudes about the product used. The first question was about the taste of the product; the second question was about the taste remaining in the mouth after rinsing; the third question was about the effect of mouthwash on the taste of food and drink; the fourth question focused on convenience in the use of mouth wash; the fifth question about the rinsing time; the last question asking for the participant observance for plaque reduction. Participants marked a point on an uncalibrated line with the negative extreme answer (0) at the left and the positive extreme answer (10) at the right end. The questionnaire and data interpreting method were adopted and modified from previous research. 43

### Statistical Analysis

#### Descriptive Statistics

Mean and median were used to describe the average values of various parameters, such as age, PI, BOP, etc... Standard deviation and variance were used to assess the data spread within each group.

#### Inferential Statistics

A one-way ANOVA (analysis of variance) test was used to compare the means of multiple groups (test, positive control, negative control) at baseline and after treatment. It helped determine if statistically significant differences existed between the groups regarding their periodontal parameters. A paired *t* -test was used to compare the means of a single group before and after treatment, assessing the effectiveness of the intervention within each group. Tukey's multiple comparisons test was used to identify specific pairwise differences between the three groups after finding significant differences with ANOVA. This helped determine which groups differed significantly from each other in terms of parameter improvements.

#### Additional Observations

The Chi-square test compares the distribution of categorical variables like gender between the groups. *p* -Values are mentioned throughout the text, indicating the level of statistical significance for each test.

## Results

### Study Population

**Fig. 3:**
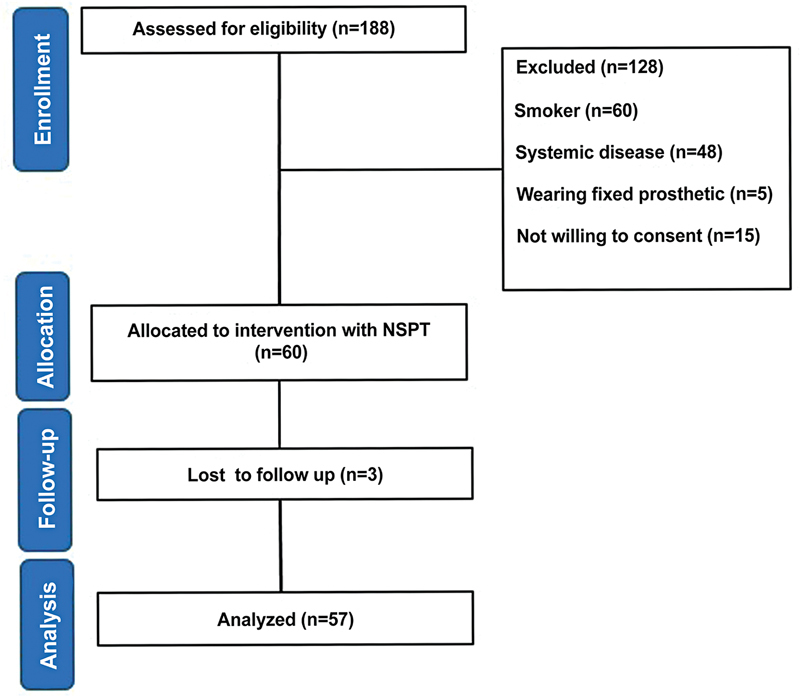
Flowchart of the present study. The diagram illustrates clinical trial design and execution.

Initially, over 100 patients diagnosed with periodontitis were examined at the clinic for eligibility according to inclusion/selection criteria. Only 60 patients were recruited in this study; they were randomly divided equally into three groups ( *n* = 20): test group (RSV), positive control group (CHX), and negative control group (placebo). One patient was excluded from the test group because of hypersensitivity to mouthwash contents, and two patients from the positive control group dropped out due to a lack of commitment to appointment dates ( Fig. 3 ).

### Age and Gender

Demographic characteristics of the study populations, including age and gender distribution, are illustrated in Table 1 . The average age for the RSV group was 37.11 ± 12.79 years, for the CHX group 36.28 ± 12.83 years, and for the placebo group 37.75 ± 9.06 years without significant difference between the groups ( *p* -value = 0.9380). Distribution according to gender showed that 12 males participated in the RSV group, 11 males in the CHX group, and 10 males in the placebo group. Seven female participants were in the RSV group, 7 females in the CHX group, and 10 female participants were in the placebo group. Gender distribution among the groups showed no significant difference ( *p* -value = 0.9269) ( Table 1 ).

**Table 1:** Demographic data of the participants at the baseline

	RSV group	CHX group	Placebo group	*p-* Value	Sig.
Total number	19	18	20		
Males	12	11	10	0.9380 a	NS
Females	7	7	10		
Age (mean ± SD), y	37.11 + 12.79	36.28 + 12.83	37.75 + 9.06	0.9269 b	NS

Abbreviations: CHX, chlorhexidine; NS, nonsignificant ( *p* > 0.05); RSV, resveratrol; SD, standard deviation.

a Comparison by Chi-square.

b Comparison by one-way ANOVA.

### Clinical Periodontal Parameters

The study's primary outcome is the reduction of periodontal PPD and BOP. Another outcome that should be considered is reducing PI and regaining clinical attachment (CAL). Clinical periodontal parameters (BOP, PPD, PI, and CAL) were assessed at the baseline visit, 7 days (first visit), and 30 days (second visit). At baseline visit, there were no significant differences in clinical periodontal parameters among the three groups ( Table 2 ). The results of the first and second visits showed a significant difference between the three groups in PI, BOP, and PPD, except CAL, which showed a nonsignificant difference between the groups ( Table 3 ).

**Table 2:** Comparison of clinical periodontal parameter scores at baseline for all interventions

		RSV group	CHX group	Placebo group	*p-* Value	Sig.
Baseline periodontal parameters	PI (mean ± SD)	88.31 ± 10.85	78.94 ± 14.03	87.37 ± 12.54	0.0515 a	NS
	BOP (mean ± SD)	58.16 ± 18.15	62.21 ± 16.76	58.96 ± 12.35	0.7160 a	NS
	PPD (mean ± SD)/mm	5.526 ± 0.513	5.222 + 0.808	5.250 ± 0.72	0.3314 a	NS
	CAL (mean ± SD)/mm	5.31 ± 1.25	6.33 ± 1.395	5.500 ± 1.539	0.0545 a	NS

Abbreviations: BOP, bleeding on probing; CAL, clinical attachment loss; CHX, chlorhexidine; NS, nonsignificant ( *p* > 0.05); PI, plaque index; PPD, probing pocket depth; RSV, resveratrol; SD, standard deviation.

a Comparison by one-way ANOVA.

**Table 3:** Descriptive and comparative statistics of periodontal parameters in first and second visits

Periodontal parameters	Visits	RSV group	CHX group	Placebo group	*p-* Value	Sig.
PI	PI first visit (mean ± SD)	15.62 ± 3.697	14.39 ± 3.010	20.37 ± 2.713	<0.0001 a	S
	PI second visit (mean ± SD)	7.274 ± 2.456	8.107 ± 2.613	17.99 ± 3.016	<0.0001 a	S
	PI first visit vs. PI second visit ( *p-* value)	<0.0001 b S	<0.0001 b S	0.0125 b S		
	PI mean difference (mean ± SD)	**8.366** ** ± 3.050**	**6.282 ± 3.374**	**2.378 ± 2.113**	** <0.0001 a**	**S**
BOP	BOP% first visit (mean ± SD)	33.36 ± 6.373	38.54 ± 6.847	41.98 ± 8.566	0.0024 a	S
	BOP% second visit (mean ± SD)	6.967 ± 1.759	10.26 ± 1.886	21.48 ± 8.006	<0.0001 a	S
	BOP first visit vs. BOP second visit ( *p-* value)	<0.0001 b S	<0.0001 b S	<0.0001 b S		
	BOP% mean difference (mean ± SD)	**26.42 ± 6.53**	**28.44 ± 6.138**	**20.55 ± 6.287**	** 0.0008 a**	**S**
PPD	PPD first visit (mean ± SD)/mm	5.105 ± 0.73	5.000 ± 0.686	5.000 ± 0.725	0.8728 a	NS
	PPD second visit (mean ± SD)/mm	3.211 ± 0.53	3.222 ± 0.64	3.850 ± 0.58	0.0014 a	S
	PPD first visit vs. PPD second visit ( *p-* value)	<0.0001 b S	<0.0001 b S	<0.0001 b S		
	PPD mean difference (mean ± SD)/mm	**1.895 ± 0.56**	**1.778 ± 0.54**	**1.150 ± 0.58**	** 0.0003 a**	**S**
CAL	CAL first visit (mean ± SD)/mm	5.316 ± 1.25	6.333 ± 1.13	5.500 ± 1.53	0.0545 a	NS
	CAL second visit (mean ± SD)/mm	4.421 ± 1.07	5.389 ± 1.09	4.900 ± 1.55	0.0765 a	NS
	CAL first visit vs. CAL second visit ( *p-* value)	<0.0001 b S	<0.0001 b S	<0.0001 b S		
	CAL mean difference (mean ± SD)/mm	**0.894 ± 0.567**	**0.944 ± 0.725**	**0.600 ± 0.502**	** 0.0998 a**	**NS**

Abbreviations: BOP, bleeding on probing; CAL, clinical attachment loss; CHX, chlorhexidine; NS, nonsignificant; PI, plaque index; PPD, probing pocket depth; RSV, resveratrol; S, significant at *p* value <0.05.

a Comparison by One-way ANOVA.

b Comparison by paired *t* -test.

Also, to show the improvement in the treatment, the periodontal parameters were compared in each group at the first and second visits, which showed significant improvement in all groups. Then the mean difference for the two visits (first and second) among the three groups was assessed; there were significant differences in all parameters ( *p* -value of BOP = 0.0008, PPD = 0.0003, PL ≤ 0.0001), except CAL ( *p* -value = 0.0998) ( Table 3 ).

**Fig. 4:**
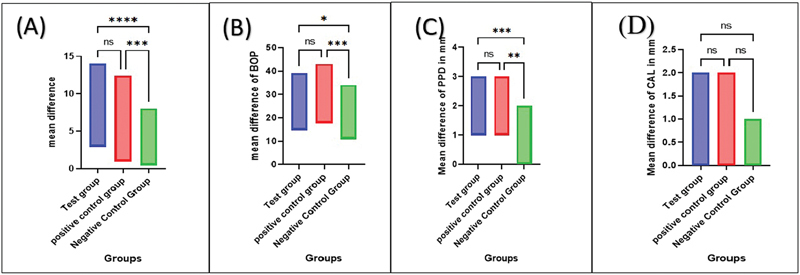
Comparisons the reduction in the mean of plaque index ( **A** ), bleeding on probing ( **B** ), probing pocket depth ( **C** ), and clinical attachment loss (CAL) ( **D** ) between three groups show nonsignificant difference between RSV group versus CHX group, while showing a significant difference between CHX versus placebo and RSV group versus placebo for all parameter except CAL which showed nonsignificant difference between the three groups. CHX, chlorhexidine; RSV, resveratrol.

Then, comparing the two groups separately showed no significant differences between the RSV and CHX groups in the four periodontal parameters ( *p* -value of BOP = 0.5971, PPD = 0.8068, PL = 0.0793, CAL = 0.8492). However, when comparing differences between the RSV group versus placebo group, significant differences were found between all the periodontal parameters ( *p* -value of BOP = 0.0148, PPD = 0.0004, PL ≤ 0.0001) except CAL ( *p* -value = 0.2681). Significant differences were also found between the CHX group versus placebo group in all the periodontal parameters ( *p* -value of BOP = 0.0009, PPD = 0.0036, PL = 0.0003) except CAL ( *p* -value = 0.0997) ( Fig. 4 ).

### Determination of Salivary Biomarker Levels Using ELISA

Salivary IL-6 and RANKL concentrations in all groups are illustrated in Table 4 . The salivary concentrations of IL-6 and RANKL showed no significant differences between the study groups at baseline visits. However, IL-6 and RANKL for each group significantly reduced between the two visits. When comparing the mean difference between the two visits, all groups showed significant differences in the IL-6 and nonsignificant in RANKL.

**Table 4:** Descriptive and comparative statistics of salivary biomarkers in the visits

Salivary biomarker	Visits	RSV group	CHX group	Placebo group	*p* -Value	Sig.
**IL-6** (pg/mL)	Baseline visit (mean ± SD)	233.6 ± 54.01	232.4 ± 60.26	205.3 ± 45.92	0.1833 a	NS
	2 ^nd^ visit (mean ± SD)	26.31 ± 7.134	26.65 ± 5.835	44.09 ± 7.894	<0.0001 a	S
	Baseline visit vs second visit (P Value)	<0.0001 b S	<0.0001 b S	<0.0001 b S		
	mean difference (mean ± SD)	207.3 ± 54.70	205.8 ± 60.80	161.2 ± 43.89	** 0.0130 a**	**S**
**RANKL** (pg/mL)	Baseline visit (mean ± SD)	55.77 ± 17.32	49.80 ± 15.90	47.68 ± 13.74	0.2603 a	NS
	2 ^nd^ visit (mean ± SD)	4.796 ± 1.728	5.114 ± 1.572	7.014 ± 3.232	0.0089 a	S
	Baseline visit vs second visit ( *P* Value)	<0.0001 b S	<0.0001 b S	<0.0001 b S		
	mean difference (mean ± SD)	50.97 ± 17.78	44.68 ± 15.96	40.66 ± 11.50	**0.1143**	**NS**

Abbreviations: CHX, chlorhexidine; IL-6, interleukin-6; NS, nonsignificant; RSV, resveratrol; S, significant at *p* value <0.05.

a Comparison by one-way ANOVA.

b Comparison by paired *t* -test.

**Fig. 5:**
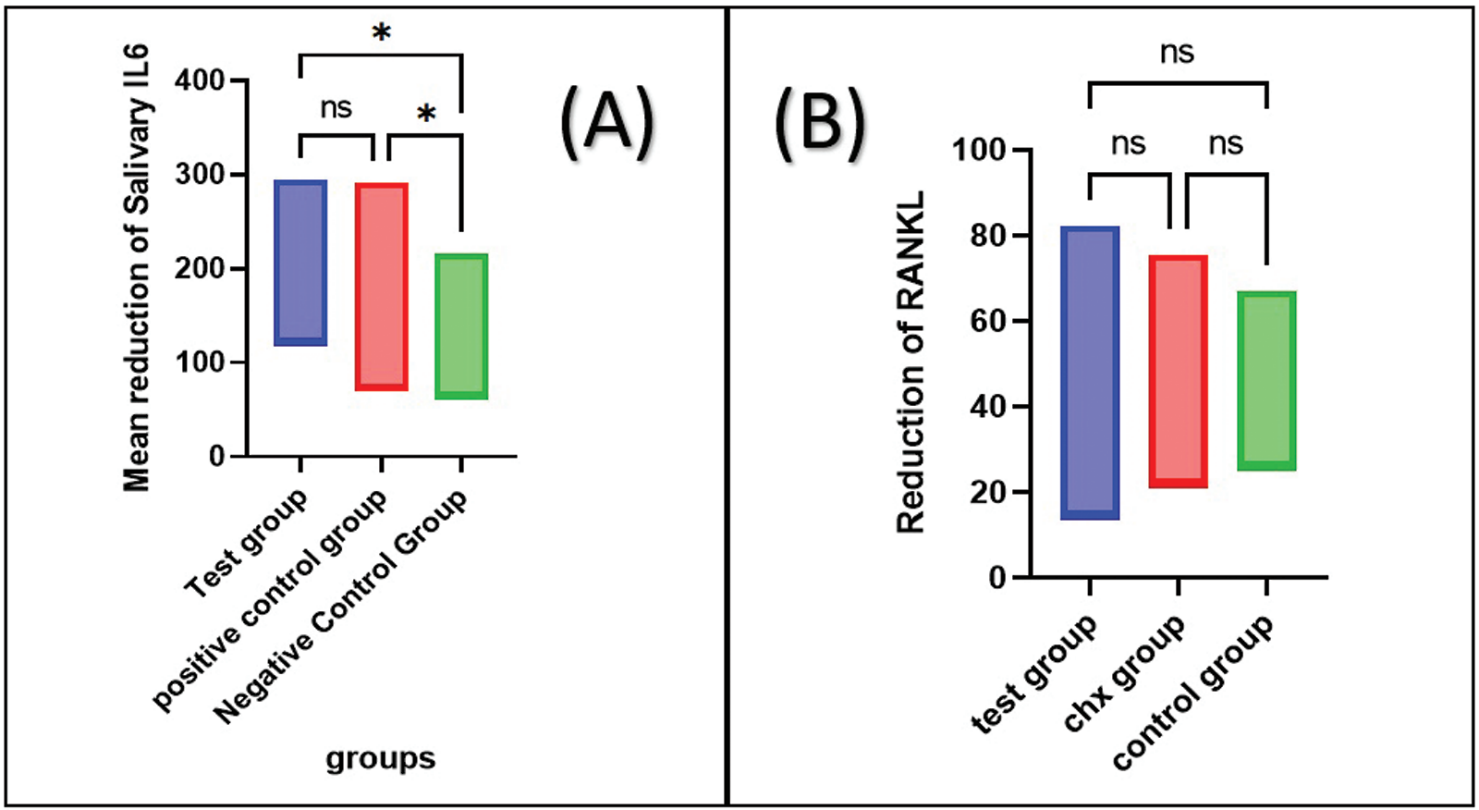
( **A** ) Comparison of the reduction in the salivary IL-6 between the three study groups showed a nonsignificant difference between RSV group versus CHX group, while a significant difference between the RSV group versus placebo group and between the CHX group versus placebo group. ( **B** ) Comparison of the reduction in the salivary RANKL between the three study groups showed nonsignificant differences between all groups. CHX, chlorhexidine; IL-6, interleukin-6.

As shown in Fig. 4 , multiple comparisons were made between the two groups separately. Regarding the comparison between the RSV and CHX groups, the Tukey test showed a nonsignificant difference in the mean reduction of IL-6 and RANKL. Comparisons between the RSV and placebo groups and between the CHX and placebo groups have shown significant differences in IL-6 but nonsignificant in RANKL ( Fig. 5 ).

### Visual Analog Scale Questionnaire

The normality test showed the participants' responses to the questionnaire, not a normal distribution, as listed in Tables 5 and 6 . Regarding participants' responses to product taste (Question 1), the participants rated the RSV mouthwash (VAS median = 5) as not having significantly better taste than the CHX (VAS median = 5.5, *p* = 0.1349) and placebo (VAS median = 5, *p* = 0.2157) mouthwashes. The length of time that the taste persists after use, as mentioned in Question 2, showed taste persistence in association with RSV mouthwash (VAS median = 7), which was not significantly higher than the CHX (VAS median = 5.5; *p* = 0.05) but was significant to the placebo (VAS median = 5; *p* ≤ 0.0001) mouthwashes. Question 3 about whether the mouthwash affects the taste of food and drinks showed no significant differences between RSV (VAS median = 5), CHX (VAS median = 5, *p* = 0.1392), and placebo (VAS median = 6, *p* = 0.3143) mouthwashes. Regarding whether the use of mouth rinse was convenient to the participant (Question 4), it was shown that the RSV mouthwash (VAS median = 8) was not different from CHX (VAS median = 7, *p* ≥ 0.9999) but was significantly higher in placebo (VAS = 8, *p* ≤ 0.0001). Responding to a rinsing time of RSV, CHX, and placebo (VAS median = 5) was significantly nondifferent ( *p* = 0.999). For the assessment of the effectiveness of mouthwashes in reducing dental plaque, the RSV and CHX have nonsignificant differences (VAS median = 6, *p* = 0.2853) but showed higher significance than placebo (VAS median = 4, *p* ≤ 0.0001)

**Table 5:** Answer to the VAS questionnaire on the mouthwashes

Question		Responses		RSV group		CHX group		Placebo group		*p-* Value	Significance
				Mean ± SD	Median	Mean ± SD	Median	Mean ± SD	Median		
Q1	How was the taste of the product?	Very bad	Very good	4.68 ± 1.05	5	5.44 ± 1.04	5.5	5.35 ± 0.93	5	0.0876	NS
Q2	How long did the taste remain in the mouth after rinsing?	Very long	Very short	6.6 ± 1	7	5.6 ± 1.1	5.5	5 ± 0.89	5	**>0.0001** a	S
Q3	How was your taste of food and drinks affected?	+ effect	− effect	5.47 ± 1.07	5	4.72 ± 0.89	5	6.10 ± 1.07	6	** 0.0014 b**	S
Q4	Was the use of mouth rinse convenient?	Not convenient	Very convenient	5.158 ± 1.12	7	5.556 ± 0.98	7	6.900 ± 0.85	8	** <0.0001 a**	S
Q5	What is your opinion about the rinsing time?	Very long	Very short	5.58 ± 1.1	6	5.67 ± 0.97	6	5.80 ± 1.06	6	0.8132	NS
Q6	What was your perception of the plaque reduction?	Insufficient	Very efficient	6.63 ± 1.12	6	5.83 ± 1.10	6	4.15 ± 1.04	4	** <0.0001 a**	S

Abbreviations: CHX, chlorhexidine; RSV, resveratrol.

Note: Kruskal–Wallis test.

a Sig. p value < 0.0001.

b Sig. *p* < 0.01.

**Table 6:** Multiple comparisons of the responses

	Q1	Q2	Q3	Q4	Q5	Q6
RSV group vs CHX group	0.1349	0.0505	0.1392	>0.9999	>0.9999	0.2853
RSV group vs. placebo group	0.2157	**<0.0001** a	0.3143	** <0.0001 a**	>0.9999	** <0.0001 a**
CHX group vs. placebo group	>0.9999	0.2623	** 0.0009 b**	** 0.0016 c**	>0.9999	** 0.0016 c**

Abbreviations: CHX, chlorhexidine; RSV, resveratrol.

Note: Dunn's multiple comparisons test.

a Sig. *p* -value < 0.0001.

b Sig. *p* -value < 0.001.

c Sig. *p* < 0.01.

## Discussion

There is a research gap on RSV mouthwash and its use as an adjunctive with supragingival PMPR and RSD. The main finding of this study was that RSV mouthwash showed improvement in the clinical periodontal parameters (PI, BOP, and PPD) and a reduction in the concentration of salivary biomarkers (IL-6 and RANKL) after 4 weeks of the baseline period. As far as we know, there has been no previous comparison between RSV and CHX mouthwash for treating periodontitis. Hence, it was not possible to verify the outcomes directly. The observations for CHX and placebo groups in this study are in concord with previous studies about the adjuvant use of the CHX, which showed a more expressive and significant improvement in clinical periodontal parameters and microbiological tests. 44 45 46

Plaque reduction is a prerequisite for controlling gingival inflammation. 47 ‏ CHX proved its antiplaque effect 48 mainly by inhibiting the growth of bacteria. 49 This study observed a significant reduction in PI measurements in RSV and CHX groups. The result of this study suggested that the antiplaque effect of RSV mouthwash is similar to the antiplaque effect of CHX mouthwash. These results agree with previous research that reported that RSV may have an inhibitory effect on the expression of cell adhesion molecules; it inhibited the endothelial dysfunction caused by *P. gingivalis* lipopolysaccharide (LPS), 50 which resulted in marked decreases in dental plaque level and gingival inflammation when using RSV due to antimicrobial effects of RSV against pathogens associated with plaque formation. 51 52 In addition, the result of this study coincides with a previous study that observed daily use of polyphenols can inhibit biofilm formation and decrease bacterial growth speed due to having important antimicrobial, antioxidant, and anti-inflammatory properties, resulting in improved clinical periodontal parameters in periodontitis. 53

Shoukheba and Elkholy also showed that RSV significantly decreased BOP scores from baseline to 6 months. 54 The improvement in bleeding of the gingiva may be related to the anti-inflammatory effect of RSV that inhibits the expression of proinflammatory cytokines, such as IL-1 and tumor necrosis factor-α, which are involved in the pathogenesis of periodontitis. 55

RSV significantly inhibited bacterial LPS. The main mechanism suggested for inhibiting LPS by RSV is the nonactivation of nuclear factor-kB. 51 In addition, RSV may trigger osteoblastogenesis, lead to new bone formation, and delay osteoclastogenesis. These features could be essential in treating periodontitis. 56 RSV may promote osteoblast differentiation to boost bone metabolism further. 57 58 However, Chin et al showed that when human gingival fibroblasts were treated with RSV for 1 hour, they showed a significant decrease in mRNA accumulation of SIRT1 and a rise in human gingival fibroblast activity, which has a significant effect on improving the periodontal pocket. 59 The results showed a significant reduction in PPD in RSV and CHX groups. This result agrees with one study that found diabetic patients with periodontitis who consumed RSV pills daily for 4 weeks had a significant decrease in mean pocket depth compared with those who consumed a placebo. 25

Means of CAL were significantly decreased at the second recall visit compared with the baseline level in all study groups, with no significant difference between them. This finding might be due to the significant effect of frequent oral hygiene instructions and NSP treatment. This result, in agreement with the previous study, showed that daily RSV supplementation for 4 weeks' adjunction with NST may not change CAL. 60 Another study observed that RSV treatments greatly enhanced the CAL in patients with periodontitis compared with placebo following an 8-week follow-up. 61 RSV prevents alveolar bone loss by attenuating the production of inflammation-related proteins, the formation of osteoclasts, and the production of circulating reactive oxygen species; in addition, RSV inhibits LPS-mediated cellular damages in human-originated gingival fibroblasts. 62 In addition to its anti-osteoclastogenesis effect, RSV may further promote osteoblast differentiation to boost bone metabolism. 57 58 The short duration of the present study's investigation might explain the controversial results regarding changes in CAL.

The key factor in the etiology of periodontitis is *P. gingivalis* , and the immune response to these microorganisms may enhance the production of inflammatory markers, including IL-6, and hard-tissue destruction. 56 RSV plays a significant antimicrobial role against the periodontal pathogens *Aggregatibacter actinomycetemcomitans* (Aa) and *P. gingivalis* (Pg). In addition, RSV exhibits anti-inflammatory properties. Its supplementation inhibited the release of proinflammatory cytokines, including IL-6. 59 The findings of this present study demonstrated a significant reduction in IL-6 concentration between baseline and the second recall visit in the RSV and CHX groups. Previous observations also found that daily RSV supplementation (as a nutritional factor in adjunct with NST) would be beneficial in improving serum levels of IL-6 in patients with periodontal disease. 60 In addition, another study found that RSV administration suppressed the high levels of IL-6 in the gingival tissue of the mice. 63 In 2014, Tamaki et al evaluated the effects of RSV supplementation on periodontal disease and detected an improvement in serum levels of IL-6 and TNF-α. 64

As well known, RANKL is pivotal in destructive bone diseases and is significantly elevated in diseased tissues by the alteration of gingival cytokine profiles. RANKL is responsible for osteoclast activation by connecting the RANK and initiating bone resorption. 65 A previous study found an increase in soluble RANKL concentration levels in individuals with periodontitis compared with healthy controls. 66 In the present study, the concentration of RANKL was significantly decreased at the second recall visit compared with the baseline level in each study group, with a nonsignificant difference between the three groups. These findings show a significant effect of NSP treatment and a nonsignificant effect of RSV and CHX on the RANKL concentration. Previous studies revealed that the therapy with RSV reduced alveolar bone loss and suggested that the presence of RSV may suppress the production of RANKL and inhibit bone collapse. 34 This result of the present study could be because the current trial's duration was insufficient to produce a noticeable effect on the concentration of RANKL.

At the end of this study, regarding the participants' feedback about using mouthwash, the first question about the product's taste regarded the unpleasant taste of RSV and CHX mouthwashes. The participants' response to taste was consistent with the results of most studies. It is well known that CHX mouthwash causes a bitter taste sensation, which could result from epithelial ion transport-based interference of CHX with specific taste buds. 67 68 In addition, many studies found that RSV was associated with bitter-tasting polyphenols. 61 RSV may activate the human bitter taste receptors TAS2R14 and TAS2R39. 69

The taste duration after use showed that the taste of RSV and CHX persisted more than the placebo. The participant's response to the duration after using CHX was consistent with the results in previous studies showing high taste persistence in association with CHX. 70

The response about the mouthwash affecting the taste of food and drinks showed a nonsignificant effect. To our knowledge, no previous study observed that RSV affects the taste of food and beverages. On the other hand, this result did not coincide with the previous research; it had been found that CHX acted on salt and bitter perceptions, progressively reducing and reaching the lowest value on the seventh day. The taste reduction lasted some days after the interruption of mouthwashes. 67 In this study, CHX did not affect the taste of foods and drinks, which may be due to the use of low-concentration products, as a previous study showed CHX concentration in mouthwashes till 0.12% and mucosa exposure not exceeding twice a day seems the best procedure to protect tastes in clinical practice. 71

Regarding whether mouth rinse was convenient for the participant, some participants in the RSV group suffered from dry mouth, and few felt itchy gingiva. Only one case suffered hypersensitivity to RSV mouth content. This study disagreed with a previous study that showed that RSV administration could increase the submandibular gland's saliva secretion and blood flow in menopausal women with salivary gland dysfunction. 72 Many studies proved the role of RSV in the treatment of xerostomia. 73 74 Therefore, the dry mouth may be caused by other contents of mouthwash.

The response to RSV, CHX, and placebo rinsing time was significantly nondifferent. Participants believed that the RSV and CHX mouthwashes effectively reduced tooth plaque. This would significantly enhance the quality of life related to oral health and positively change the patients' perception long after the therapy has terminated. 75 76

There are some limitations in the present study, such as the short study duration causing insufficient time to produce a noticeable effect on the clinical parameters such as CAL and the concentration of biomarkers such as RANKL, also the use of saliva as a source of biomarkers may affect the measurement due to the dilution effect and lack of site-specific information, which is better evaluated by using gingival crevicular fluid (GCF).

## Conclusion

RSV -containing mouthwash significantly affects the treatment of periodontitis when used as an adjunct to nonsurgical periodontal therapy. RSV showed a significant reduction in the clinical periodontal parameters PI, BOP, and PPD and a noticeable reduction of salivary IL-6 concentration. So, RSV mouthwash can be used as an alternative to CHX mouthwash for patients with periodontitis.
